# Structural insights into selective inhibition of leishmanial GDP-mannose pyrophosphorylase

**DOI:** 10.1038/s41421-022-00424-z

**Published:** 2022-08-30

**Authors:** Hang Li, Tuo Ji, Qi Sun, Yao Chen, Weiya Xu, Chengdong Huang

**Affiliations:** 1 Ministry of Education Key Laboratory for Membraneless Organelles & Cellular Dynamics, School of Life Sciences, Division of Life Sciences and Medicine, University of Science and Technology of China, Hefei, Anhui China; 2 Jiangxi Key Laboratory of Organic Chemistry, Jiangxi Science and Technology Normal University, Nanchang, Jiangxi China

**Keywords:** Cryoelectron microscopy, Molecular biology

Dear Editor,

Leishmaniasis is a neglected tropical disease (NTD) caused by infection with the protozoan parasite *Leishmania*. The World Health Organization (WHO) estimates nowadays leishmaniasis threatens 310 million people in 98 countries with approximately one million new cases reported each year^1^, causing annual mortality of about 60,000 worldwide^2^. Existing therapies suggested by the WHO are, however, limited to a handful of drugs^3^, which have demonstrated severe issues of toxicity and/or drug resistance^4^. In this context, development of new effective antileishmanial drugs is a global health priority.

A prominent therapeutic target for antileishmanial drug development is leishmanial GDP-mannose pyrophosphorylase (GDP-MP or GMPP, EC 2.7.7.13), an enzyme catalyzing the formation of GDP-mannose by condensation of mannose-1-phosphate (Man-1-P) and GTP (Supplementary Fig. S1a). As GDP-mannose serves as a central precursor for biosynthesis of diverse glycoconjugates necessary for host–parasite recognition and is essential for parasite survival, disruption of *Leishmania* GDP-MP activity leads to complete loss of virulence, suggesting this enzyme as an attractive target for development of new drugs against leishmaniasis^5^. However, lack of experimentally resolved structures of this protozoan enzyme has greatly impeded structure-based drug design.

Here we recombinantly expressed and purified this enzyme from *L. donovani* (LdGDP-MP) (Supplementary Fig. S1b), the leishmanial species found in Africa and Asia that causes visceral leishmaniasis (also known as kala-azar, ‘black fever’), the most severe form of leishmaniasis, and assessed the oligomerization status by size-exclusion chromatography coupled to both multi-angle light scattering and quasi-elastic light scattering. LdGDP-MP exhibits a hexameric structure with a molecular weight of ~245.3 kDa (Supplementary Fig. S1c), consistent with previous observations^6^. The enzymatic properties of LdGDP-MP are described in Supplementary Data S1 and Figs. S1, S2.

We next solved the structure of LdGDP-MP in the absence of any cofactor or substrate at the resolution of 3.4 Å using single-particle cryo-EM imaging technique, which comprises of six subunits and exhibits a D3 symmetry with a dimension of ~104 Å × ~104 Å × ~108 Å (Fig. 1a). Each subunit is made of two separate domains, a N-terminal Rossmann-like fold commonly observed in nucleotidyltransferases and many other nucleotide-binding proteins, and a C-terminal left-handed β-helix domain containing tandem repeats of a [I/L/V]-G-X(_4_) hexapeptide motif (Fig. 1b), a characteristic structure found in the transferase hexapeptide repeat family^7^. The central twisted β-sheet in the N-terminal domain is made of nine β-strands, which are arranged in the order of 3-2-1-4-9-10-6-7-8 and flanked by six α-helices and a pair of antiparallel β-strands (Supplementary Fig. S4).

**Fig. 1:**
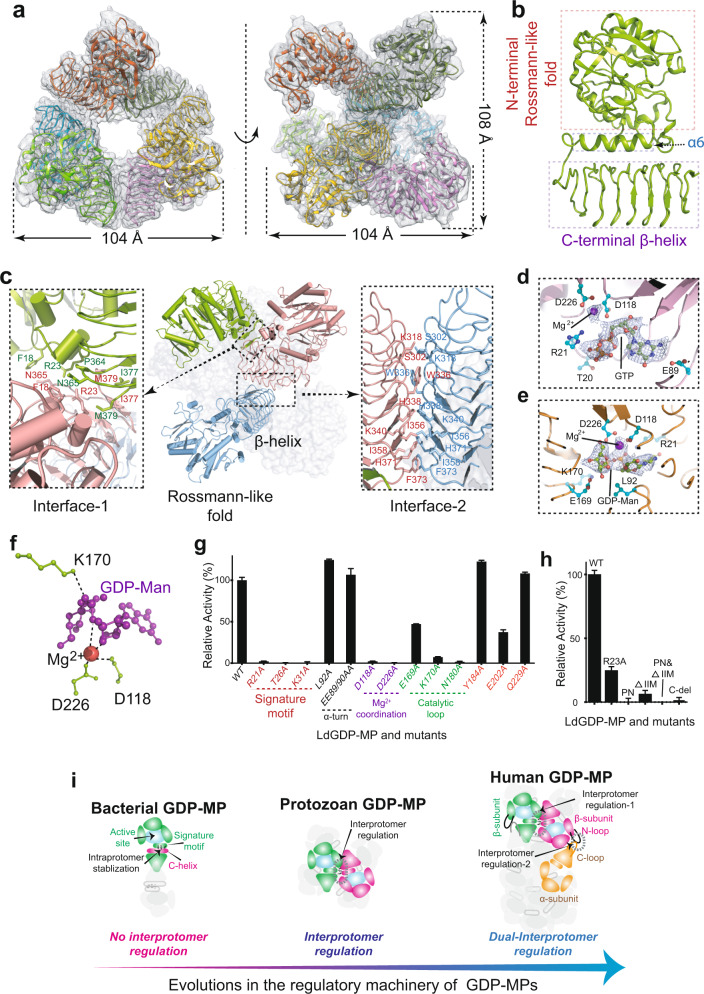
Cryo-EM and mechanistic studies of LdGDP-MP. **a** Overall views of hexameric LdGDP-MP with six subunits colored differently. The real density maps are shown in grey mesh. **b** Ribbon diagrams of a LdGDP-MP monomer. **c** Detailed interface contacts between neighboring subunits. **d**, **e** Magnified views of GTP- (**d**) or GDP-Man- (**e**) binding pocket with the real density map of GTP or GDP-Man shown in mesh. **f** Catalytic center of LdGDP-MP. **g** Activity comparisons of LdGDP-MP with mutants as labeled. **h** Impacts on the enzyme activity upon mutations for disrupting the interface-1 of LdGDP-MP. **i** Proposed evolutionary pathway of GDP-MPs from bacteria to human in terms of activity modulation. All enzyme assays were repeated at least three times and data were shown as means ± SD.

The homoheximeric LdGDP-MP complex is organized as a dimer of trimers, with each subunit pinched by two neighboring subunits via two interacting regions, namely, interface-1 and interface-2 (Fig. 1c; Supplementary Fig. S5). In interface-1, the two subunits are attached in a shape-complementary manner between the region around the canonical signature motif GGXGXR(L)XPLX_5_PK of pyrophosphorylases (PPases)^8^ in one protomer and the C-terminus of another protomer; whereas interface-2 formation is mediated exclusively by the two C-terminal domains crossed at ~70 degrees. The contact surfaces for interface-1 and interface-2 amount to ~590 Å^2^ and ~750 Å^2^, respectively; and the interactions are established predominantly by hydrophobic interactions together with a few hydrogen bonds (Supplementary Fig. S5b, d).

To gain insights into the catalytic mechanism of leishmanial GDP-MP, we further solved the cryo-EM structures of LdGDP-MP in complex with its substrate, GTP, or the reaction product, GDP-mannose at the resolution of 3.0 Å, or 3.2 Å, respectively (Supplementary Figs. S6, S7). These structures clearly mark a closed and well-ordered active pocket with approximate dimensions of 14 Å × 15 Å × 17 Å, delimited by β1–β4–β9 in the central β-sheets as the pocket base (Fig. 1d, e; Supplementary Fig. S8a, b). The pocket side walls are shaped by a large amount of loops or α-turns. Among these pocket-defining components, the β8–β9 linking loop (residues 169–182) comprises the F(V)EKP motif that has been described as part of the GDP-MP active site^8–10^, and is thereby denoted as the catalytic loop.

Structural comparisons reveal that binding to GTP or GDP-mannose induces significant conformational changes primarily for the loop regions surrounding the active site, and results in an overall more compact conformation (Supplementary Fig. S8c). Indeed, the key lysine residue (K170) in the F(V)EKP motif shifts ~5 Å or ~8 Å towards the catalytic center upon GTP or GDP-mannose binding. The catalytically essential Mg^2+^ is coordinated by two absolutely conserved aspartate residues, D118 and D226. In the structure of GTP-bound LdGDP-MP, the guanidine moiety is sandwiched between the α2–α3 loop and the loop connecting the α1 and β1. Selective recognition of the guanidine purine ring is attained by interactions of the exocyclic amino group with E89 and G96, by interactions of N1 with the side-chain of E88, and by interactions of the guanidine carbonyl with the main-chain nitrogen atoms of E90. The phosphate groups are primarily anchored by the signature motif especially the absolutely conserved residue R21, in addition to Mg^2+^. In the GDP-mannose-bound LdGDP-MP, the guanidine moiety, compared to that in the GTP-bound form, scrunches ~4 Å from the cleft end towards the catalysis center. Intriguingly, the GDP-mannose molecule adopts a seemingly unfavorable V-shape conformation (Fig. 1e), which may facilitate the condensation reaction and subsequent product release. The sugar moiety is secured by the close contact of the hydroxyls with the central β-sheet amino acids, as well as the Mg^2+^-coordinating residue D226. In particular, the K170 side-chain in the F(V)EKP motif inserts deeply into the catalytic center to make close contact with the phosphate group next to the sugar moiety (Fig. 1f), indicative of its direct involvement in catalysis. The detailed interactions of LdGDP-MP with its substrate/product are shown in Supplementary Fig. S9.

It is noteworthy that we failed to obtain the structure of LdGDP-MP in complex with Man-1-P. The inability of Man-1-P in forming a stable complex with LdGDP-MP in absence of GTP readily implies the sequentially ordered Bi-Bi mechanism. Taken together, our data support that the reaction catalyzed by LdGDP-MP is the S_N_2 type with GTP binding to the enzyme first, followed by binding of Man-1-P, which acts as the nucleophile attacking the α-phosphate of GTP. The β- and γ-phosphates of GTP are then displaced and leave as pyrophosphate. In this scenario, the attacking nucleophile (Man-1-P) and the leaving group (pyrophosphate) reside at opposite sides of the central reaction atom, the phosphorus atom of the GTP α-phosphate, which is consistent with the structural data presented here (Fig. 1d, e). The mechanisms proposed above have been validated by mutagenesis, and the results are shown in Fig. 1g and described in Supplementary, Data S1.

To date, three GDP-MP structures from different species have been experimentally resolved: the bacterial TmGDP-MP^8^, human GDP-MP^10^, and the protozoan LdGDP-MP presented here (Supplementary Fig. S10a–c). Structural comparisons are discussed in details in Supplementary Data S1. The striking structure difference between the prokaryotic and eukaryotic GDP-MPs leads to distinct interprotomer contact patterns: while the two subunits in prokaryotic GDP-MP are attached exclusively via the C-terminal β-helix domains, the eukaryotic GDP-MP enzymes contain an extra interprotomer interaction interface (interface-1 shown in Fig. 1c). The function of this extra interprotomer communication evolved in eukaryotic GDP-MP enzymes remains unknown.

We, therefore, made a series of mutations to interrupt the interactions in interface-1 (Supplementary Fig. S13) and evaluated the mutational impacts. Strikingly, replacement of R^23^ with alanine (R23A) caused loss of ~77% activity, whereas the mutations of P^364^N^365^ to arginines (PN), deletion of the last three residues on the C-terminus (ΔIIM), their combination (PN&ΔIIM), or deletion of the entire C-terminal domain (C-del) rendered the enzyme completely inactive (Fig. 1h), indicating the essential role of interprotomer communication in attaining leishmanial GDP-MP activity. As expected, these mutations exert no noticeable impact on the folding properties of enzymes, as all mutants demonstrate properly folded circular dichroism spectra (Supplementary Fig. S14a). On the other hand, disruption of the interface-1 contacts led to instability of the quaternary organization, as evidenced by drastic changes in the oligomerization status (Supplementary Fig. S14b–e). In support of this, thermal shift assays showed that mutations on the interface-1 significantly lowered melting temperatures (T_m_) (Supplementary Fig. S14f, g).

Considering that part of the highly conserved signature motif is directly involved in the interprotomer contacts (Supplementary Fig. S13), we infer that the interprotomer interactions presented in the interface-1 secure the signature motif, which is critical for substrate anchoring (Fig. 1d), and thus provides stabilized platform necessary for catalysis.

Finally, we looked at the regulatory machineries of GDP-MPs in terms of evolutionary transition and attempted to unveil the transition route from the homodimeric bacterial GDP-MP to the structurally more complex heterododecameric human enzyme (Fig. 1i). In the dimeric TmGDP-MP, the two subunits are attached solely via the C-terminal domain. Therefore, each bacterial subunit functions largely as an independent entity, and the signature motif, which is critical for substrate coordination, is conformationally stabilized by a C-terminal helix (C-helix) residing at the interface between the N- and C-terminal domains (Supplementary Fig. S10d). Eukaryotic GDP-MPs lack the C-helix, and the signature motif is functionally secured by interprotomer contacts instead, which, in turn, build a structurally more sophisticated enzyme edifice, as presented in the hexameric protozoan GDP-MP or the heterododecameric human GDP-MP. In the supramolecular human GDP-MP enzyme, the presence of inactive α-subunits offers additional interprotomer contacts between the N-loop in the β-subunit and the so-called “C-loop” in the α-subunit, and such interactions have been shown to play an allosteric role in negative regulation of activity^10^. In this scenario, human GDP-MP possesses two distinct sets of interprotomer communications (“dual-interprotomer regulation”) and both are involved in activity regulation, either positively or negatively, for fine-tuning of the enzymatic action (Fig. 1i).

As such, we reveal a stepwise evolutionary scenario about how the regulation machinery gradually advances from a relatively simple mode for the bacterial GDP-MP to a sophisticated one for the human enzyme (Fig. 1i). Although the reason that various GDP-MPs adopt distinct oligomeric forms is far from obvious, we propose here the extra controls that enable the assembling of supramolecular eukaryotic GDP-MPs with extra interprotomer contacts offer a more robust avenue for activity modulation, presumably to better meet the ever-changing metabolic needs that are often faced by eukaryotes. The difference in regulatory mechanisms of *Leishmania* and human GDP-MPs revealed here may provide useful insights into antileishmanial drug design.

## Supplementary information

Supplementary information
