# Supplementary material for: Structural insights into selective inhibition of leishmanial GDP-mannose pyrophosphorylase
Source: Cell Discov. 2022 Aug 30;8:83. doi: 10.1038/s41421-022-00424-z (PMC9424295; doi:10.1038/s41421-022-00424-z)
Supplement: Supplementary file 1 — Supplementary information [file 41421_2022_424_MOESM1_ESM.pdf]

**Supplementary data S1**

**Enzymatic characterization of LdGDP-MP**

We characterized the enzymatic properties of LdGDP-MP. Steady-state kinetic measurements yielded turnover numbers ($k_{cat}$) and Michaelis constants ($K_m$) for Man-1-P of ~266 $min^{-1}$ and ~45 μM, respectively; or for GTP with the corresponding values of ~208 $min^{-1}$ and ~42 μM, respectively (Fig. S1d-e). The activity of LdGDP-MP is strictly dependent on the presence of divalent metal ion cofactor of $Mg^{2+}$ (Fig. S2b), which demonstrates the maximal activity with the concentration above 10 μM (Fig. S2c) at the pH value of ~ 8 (Fig. S2d). LdGDP-MP exhibits high substrate specificity as the presence of glucose-1-phosphate or ATP fails to invoke the catalysis activity (Fig. S2e). Interestingly, under the experimental conditions tested, LdGDP-MP behaves as a thermophilic enzyme, as it demonstrates optimal activity above 55 °C (Fig. S2f). Nevertheless, we decided to perform all following enzyme assays at the temperature of 37 °C to maintain the assay temperature consistent with that in host cells.

**Catalytic mechanism validation**

To validate the mechanism proposed above, we performed a series of mutagenesis to the key residues identified above and evaluated the mutational effects on catalytic activity. Mutations of the signature motif residues (R21, T26 or K31) involved in substrate anchoring, the $Mg^{2+}$ coordination residues (D118 or D226), residues within the catalytic loop (E169, K170 or N180 that captures the sugar moiety), or the residue E202 completely abolished or severely diminished activity (Fig. 1g). Interestingly, replacement of the α-turn residues (L92, E98/E99), Y184 or Q229 with alanine showed only subtle changes in enzymatic activity, indicative of insignificant role played by these residues in catalytic

process (Fig. 1g). Indeed, such sequential ordered $S_N2$ type catalytic mechanism is evolutionally conserved for other NDP-sugar pyrophosphorylases [1].

## Structural comparisons of GDP-MPs from different species

Structural alignment GDP-MPs from various species reveals that the bacterial enzyme is conserved with its eukaryotic counterparts to only a moderate extent, as evidenced by the r.m.s.d. of ~3.2 Å for alignment of the catalytic N-terminal domains (~6.0 Å for the full-length protein alignment). The C-terminal domain in the bacterial GDP-MP is significantly shorter than its eukaryotic counterparts with an orientation deviated ~ 23° clockwise comparing to the LdGDP-MP (Fig. S10d). In contrast, the structures of the leishmanial and human GDP-MPs are very similar to each other (Fig. S10e), as highlighted by the r.m.s.d. value of ~0.9 Å. The conserved amino acids are especially clustered around the catalytic pocket, while the peripheral regions and the C-terminal domain are less conserved (Fig. S12). Notably, two loop regions, residues 142–150 (N-loop) and the catalytic loop (residues 137–142 and residues 159–165 in human GDP-MP β-subunit), demonstrate considerable structural variations between LdGDP-MP and human GDP-MP (Fig. S10e and Fig. S12b). Considering the key role of the catalytic loop in activity modulation and the role of N-loop region in allosteric regulation [2], such structural variations might provide opportunity for future antileishmanial drug development.

## Methods and Materials

Cloning, expression and purification

The target gene encoding LdGDP-MP from *Leishmania donovani* was custom synthesized and cloned into the pET28a-sumo vector, then transformed into *E. coli* BL21(DE3) cells. For expressing His-sumo-LdGDP-MP, BL21(DE3) cells were cultured in Luria Bertani (LB) medium supplemented with Kanamycin (50ug/ml) at 37 °C until OD600 nm reached ~0.6. Cells were induced by adding isopropyl-β-D-1-thiogalactopyranoside (ITPG) to the final concentration of 0.3 mM, and incubated for 16 h at 18 °C. Cells were harvested by centrifugation at 5,000g for 20 mins and resuspended in lysis buffer (25 mM Tris-HCl, pH 8.0, 500 mM NaCl, 10 mM imidazole and 1 mM PMSF).

Cells were broken in a high-pressure homogenizer (JN-Mini Pro Low-temperature Ultra-high-pressure cell disrupter, JNBIO) and subjected to centrifugation at 50,000g for 30 mins at 4°C. The supernatant was applied to HisTrap FF column (GE Healthcare), and the bound proteins were eluted with an elution buffer (25 mM Tris-HCl, pH8.0, 150 mM NaCl and 300 mM imidazole). The eluted proteins were treated with SUMO protease for Sumo tag cleavage at 4°C while dialyzing against dialysis buffer (50 mM Tris-HCl pH8.0, 100 mM NaCl, 3 mM β-mercaptoethanol) overnight. The dialyzed protein samples were applied to HisTrap FF column and further purified by Hiload 16/600 Superdex 200 column (GE Healthcare) in 50 mM Tris-HCl, pH7.5, 100 mM NaCl, 1 mM DTT, and 8 mM $MgCl_2$. The purity of proteins was assessed by SDS-PAGE.

SEC-MALS (Multi-angle light scattering)

LdGDP-MP or mutants (monomer concentration in the range of 30-50 μM) in 50 mM Tris-HCl, pH7.5, 100 mM NaCl, 1 mM DTT, and 8 mM $MgCl_2$ were injected into the

Superdex 200 increase 10/300 GL column (GE Healthcare) connected to DAWN HELEOS II combined Optilab TrEX system (Wyatt Technology Corporation). Molecular weight was analyzed using the program of ASTRA version7.0.1 (Wyatt Technology Corporation).

<u>LdGDP-MP activity assay</u>

The activity assays for LdGDP-MP and mutants were performed using a colorimetric assay coupled with inorganic pyrophosphatase [3,4]. Briefly, the reaction was carried out at 37 ℃ for 6 mins in 50 mM Tris-HCl, pH7.5, 100 mM NaCl, 1 mM DTT, and 8 mM $MgCl_2$, containing 2 ng/μL of enzyme, 150 μM of Mannose-1-P, 0.4 units $ml^{-1}$ inorganic pyrophosphatase (New England Biolabs) and 150 μM GTP. Steady-state kinetic parameters were determined by double reciprocal Lineweaver-Burk plot for reactions that followed Michaelis-Menten kinetics. Same protocol with various concentrations of GTP from 5 to 150 μM, or various concentrations of Mannose-1-P from 5 to 150 μM was applied to determine the kinetic parameters of LdGDP-MP for GTP or Mannose-1-P.

<u>Circular dichroism measurement</u>

The protein samples (5 μM in 50 mM Tris-HCl, pH7.5, 100 mM NaCl, 1 mM DTT, and 8 mM $MgCl_2$) were recorded by a Chirascan qCD spectrometer (Applied Photophysics Ltd.) with the following experiment parameters: bandwidth 1.0 nm; wavelength coverage 180-260 nm; step 1 nm; time per point 0.25 s; repeat 10.

<u>Thermal Shift Assays</u>

Reaction mixtures containing 2 μM LdGDP-MP (monomer concentration) or its mutants, and 5× SYPRO Orange protein gel stain (Sigma) in 50 mM Tris-HCl, pH7.5, 100 mM NaCl, 1 mM DTT, and 8 mM $MgCl_2$ were prepared in a 96-well PCR plate. Plate was incubated

in CFX connect real-time system (Bio-Rad) at 20℃ for 10s, then heated to 98℃ at a rate of 0.2℃/10s with FRET channel reading. Melting temperatures were calculated by the minimum point of the –d(RFU)/dT curve which recorded and exported by Bio-Rad CFX Maestro.

Single-particle cryo-electron microscopy data collection

For cryo-EM, 3 µL of protein samples (0.1 mg/mL Apo-LdGDP-MP; 0.5 mg/mL LdGDP-MP with 2 mM GTP; 1.0 mg/mL LdGDP-MP with 0.5 mM GDP-Mannose and 0.002% DDM) were applied on glow-discharged Quantifoil R1.2/1.3 100 holey-carbon Co grids pre-treated with poly-L-Lysine using a Vitrobot Mark IV (ThermoFisher Scientific). The samples were first screened on a FEI TF20 operated at 200 keV. Images of Apo-LdGDP-MP were collected using Titan Krios G3i microscope (FEI) operated at 300 kV with a Gatan K2 Summit direct detection camera. The data were acquired using the SerialEM in super-resolution mode with a nominal magnification of 29,000x, yielding a pixel sizes of 0.505 Å with a total dose of 51 e/ $Å^2$. The defocus ranges were set from −1.6 µm to −2.3 µm. Images of other samples were collected with Gatan BioQuantum K3 camera and acquired using the Thermo Scientific EPU 2.10 in counting mode with a nominal magnification of 29,000x, yielding a pixel sizes of 1.07 Å with a total dose of 51 e/ $Å^2$.

Cryo-electron microscopy image processing, 3D reconstruction and analysis

cryoSPARC was used for all processing steps. A total of 3154 raw movie stacks were acquired for Apo-LdGDP-MP (6505 raw movie stacks for LdGDP-MP in complex with GTP, and 4951 raw movie stacks for LdGDP-MP+GDP-Mannose) and subjected to patch motion correction and patch CTF estimation in cryoSPARC. About 500 particles were manually picked to generate templates for auto-picking. The reference-free 2D

classification was conducted with the auto-picked particles and the protein particles were extracted by a box size of 256 pixel. After particle screening using 2D and 3D classification, the final 56,825 particles for Apo-LdGDP-MP, 271,350 particles for LdGDP-MP-GTP, and 123,461 particles for LdGDP-MP-GDP-Mannose were subjected to Ab-Initio Reconstitution. We initially processed the data with no symmetry imposed, and the structure solved exhibits a D3 symmetry as analyzed by AnAnaS with the symmetry group root-mean-square deviation (r.m.s.d.) of ~0.42 Å [5]. We therefore applied D3 symmetry and solved the LdGDP-MP structure. The final resolutions for apo-LdGDP-MP, LdGDP-MP-GTP complex and LdGDP-MP-GDP-Mannose complex were 3.4 Å, 3.04 Å, and 3.16 Å respectively, as estimated based on the gold-standard Fourier shell correlation (FSC) using the 0.143 criterion (Fig. S15).

Model building and structural refinement

Initial model of LdGDP-MP was generated using GMPPA/GMPPB structure (PDB 7d72) as a reference and docked into the cryoEM maps using UCSF Chimera. The sequences were mutated with corresponding residues in LdGDP-MP, followed by rebuilding by the program of Coot. The missing residues were not built due to the lack of corresponding densities. Real-space refinement of models with geometry and secondary structure restraints applied was performed using PHENIX. The final model was refined and validated by PHENIX with the cryoEM maps. The statistics of cryo-EM data collection, refinement and model validation are summarized in Table S1. All structural data have deposited in the Protein Data Bank (PDB) and the Electron Microscopy Data Bank (EMDB) under the following accession codes: Apo LdGDP-MP, PDB 7WHR (EMDB 32509);

LdGDP-MP in complex with GTP, PDB 7WHS (EMDB 32510); and LdGDP-MP in complex with GDP-Man, PDB 7WHT (EMDB 32511).

## References:


1 Kim, H. *et al.* Structural basis for the reaction mechanism of UDP-glucose pyrophosphorylase. *Mol Cells* **29**, 397-405, doi:10.1007/s10059-010-0047-6 (2010).

2 Zheng, L. *et al.* Cryo-EM structures of human GMPPA-GMPPB complex reveal how cells maintain GDP-mannose homeostasis. *Nat Struct Mol Biol* **28**, 1-12, doi:10.1038/s41594-021-00591-9 (2021).

3 Davis, A. J. *et al.* Properties of GDP-mannose pyrophosphorylase, a critical enzyme and drug target in Leishmania mexicana. *J Biol Chem* **279**, 12462-12468, doi:10.1074/jbc.M312365200 (2004).

4 Chen, Y. *et al.* Assembly status transition offers an avenue for activity modulation of a supramolecular enzyme. *Elife* **10**, doi:10.7554/eLife.72535 (2021).

5 Pages, G. & Grudinin, S. Analytical symmetry detection in protein assemblies. II. Dihedral and cubic symmetries. *J Struct Biol* **203**, 185-194, doi:10.1016/j.jsb.2018.05.005 (2018).

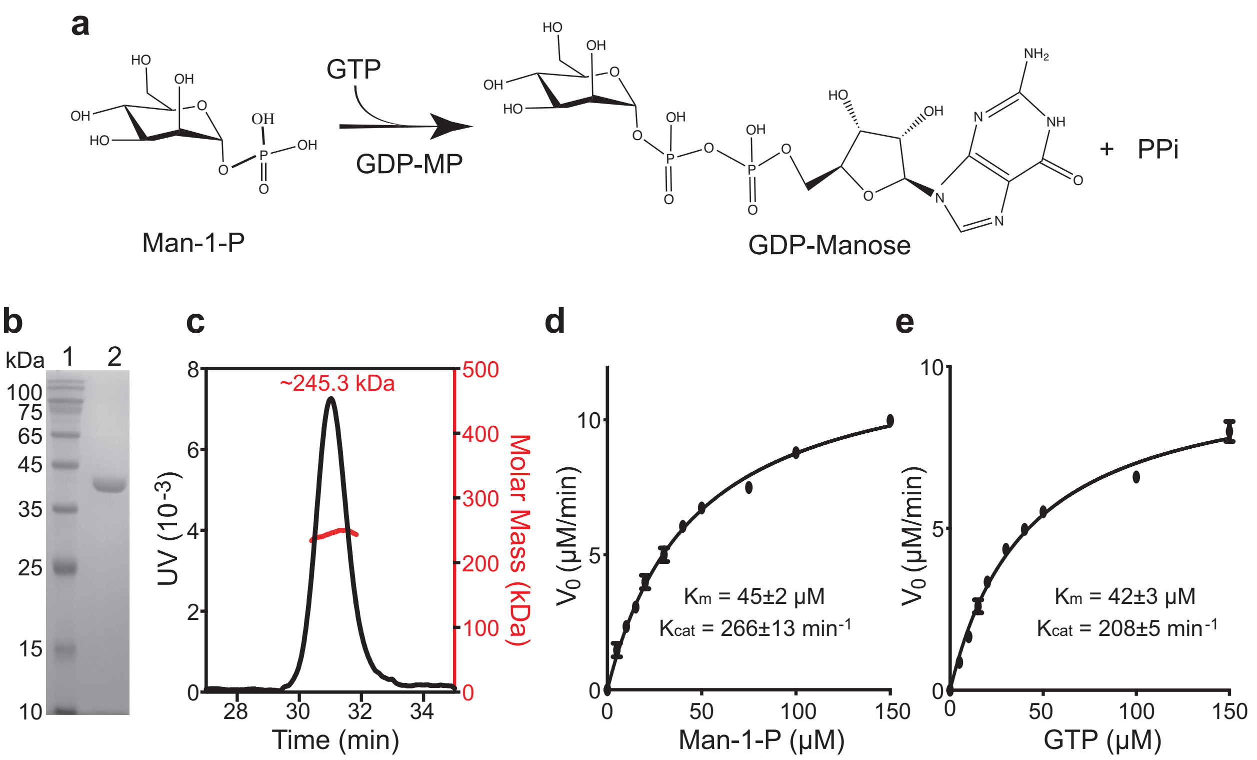
a
GTP
GDP-MP
Man-1-P
GDP-Manose
+ PPi
b
kDa 1 2
100
75
65
45
35
25
15
10
c
~245.3 kDa
UV (10-3)
Molar Mass (kDa)
Time (min)
d
V0 (μM/min)
Km = 45±2 μM
Kcat = 266±13 min-1
Man-1-P (μM)
e
V0 (μM/min)
Km = 42±3 μM
Kcat = 208±5 min-1
GTP (μM)

Fig. S1

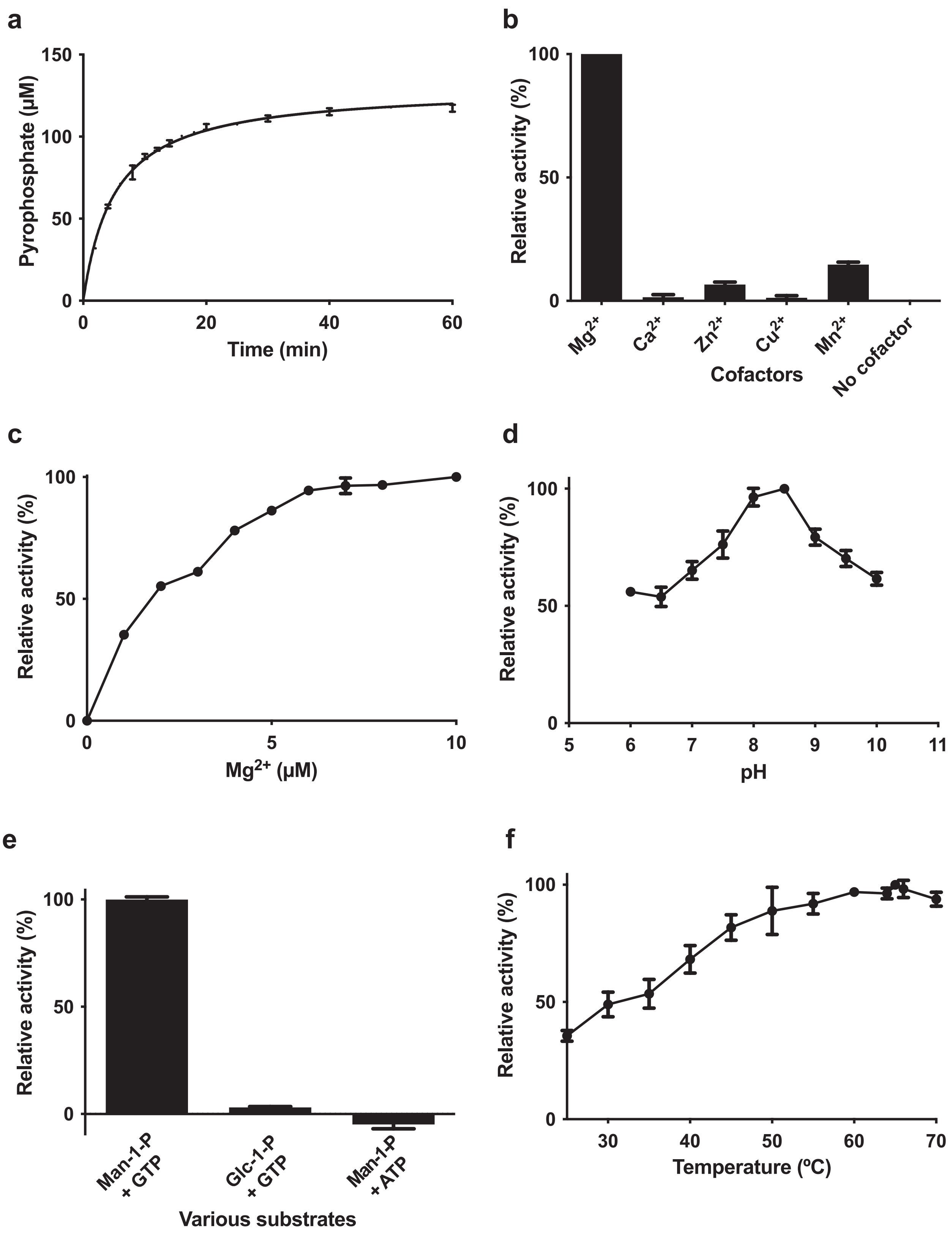

a
Pyrophosphate (μM)
Time (min)
b
Relative activity (%)
Mg2+
Ca2+
Zn2+
Cu2+
Mn2+
No cofactor
Cofactors
c
Relative activity (%)
Mg2+ (μM)
d
Relative activity (%)
pH
e
Relative activity (%)
Man-1-P + GTP
Glc-1-P + GTP
Man-1-P + ATP
Various substrates
f
Relative activity (%)
Temperature (ºC)


Fig S2

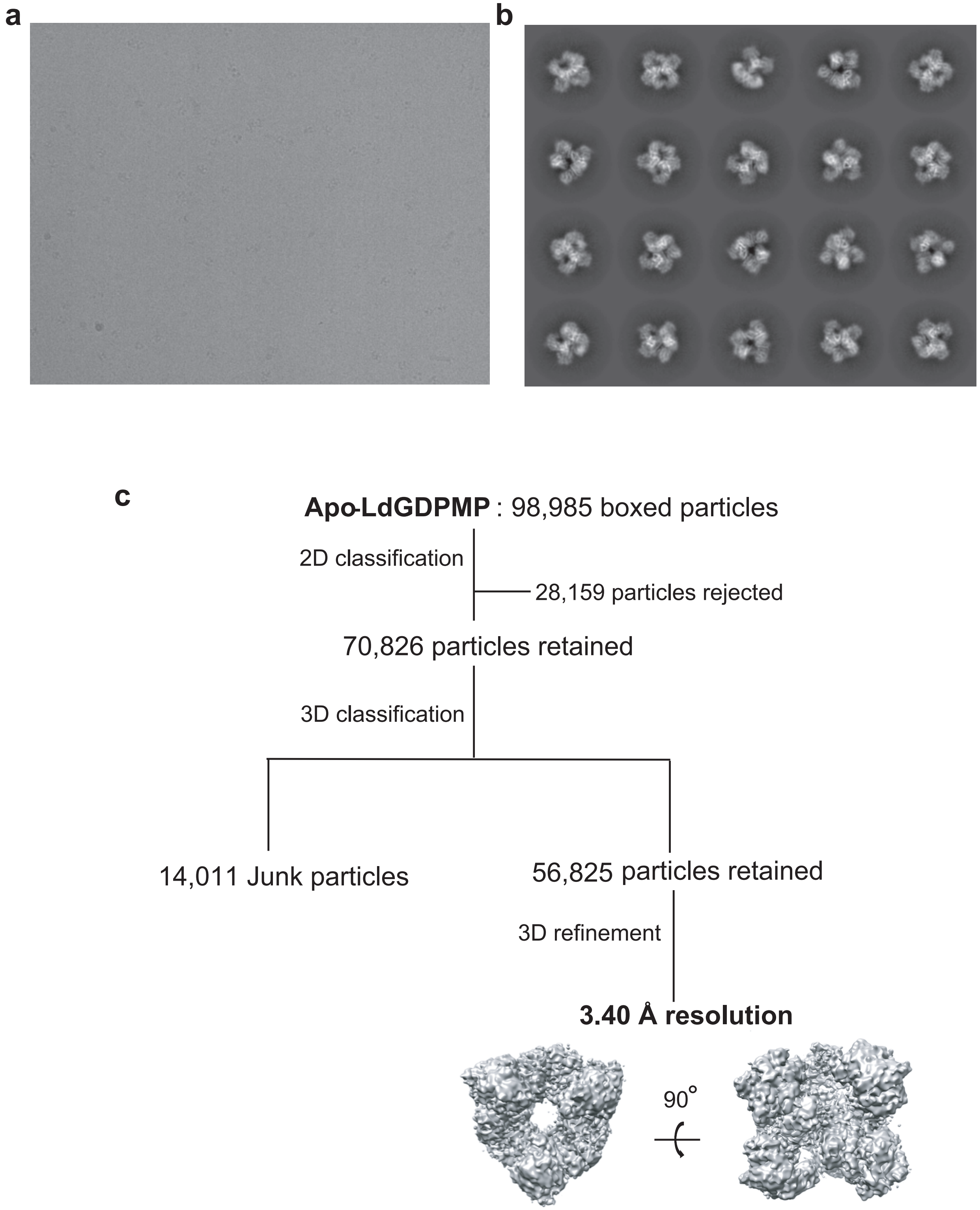
a
b
c
Apo-LdGDPMP : 98,985 boxed particles
2D classification
28,159 particles rejected
70,826 particles retained
3D classification
14,011 Junk particles
56,825 particles retained
3D refinement
3.40 Å resolution
90°

Fig. S3

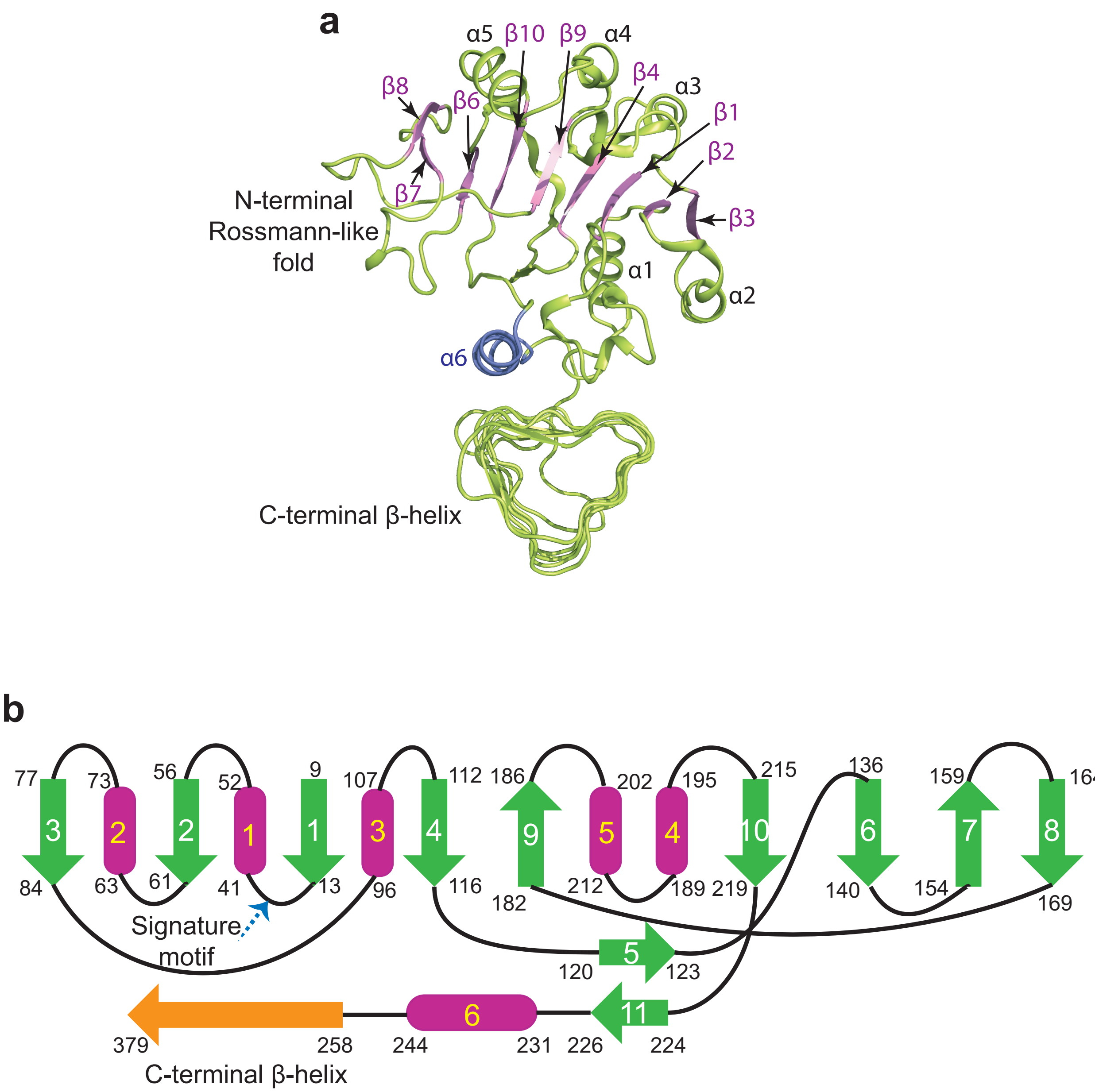

a
α5
β10
β9
α4
β4
α3
β8
β6
β1
β2
β7
β3
N-terminal Rossmann-like fold
α1
α2
α6
C-terminal β-helix
b
77 73 56 52 9 107 112 186 202 195 215 136 159 164
84 63 61 41 13 96 116 182 212 189 219 140 154 169
Signature motif
120 123
379 258 244 231 226 224
C-terminal β-helix


Fig. S4

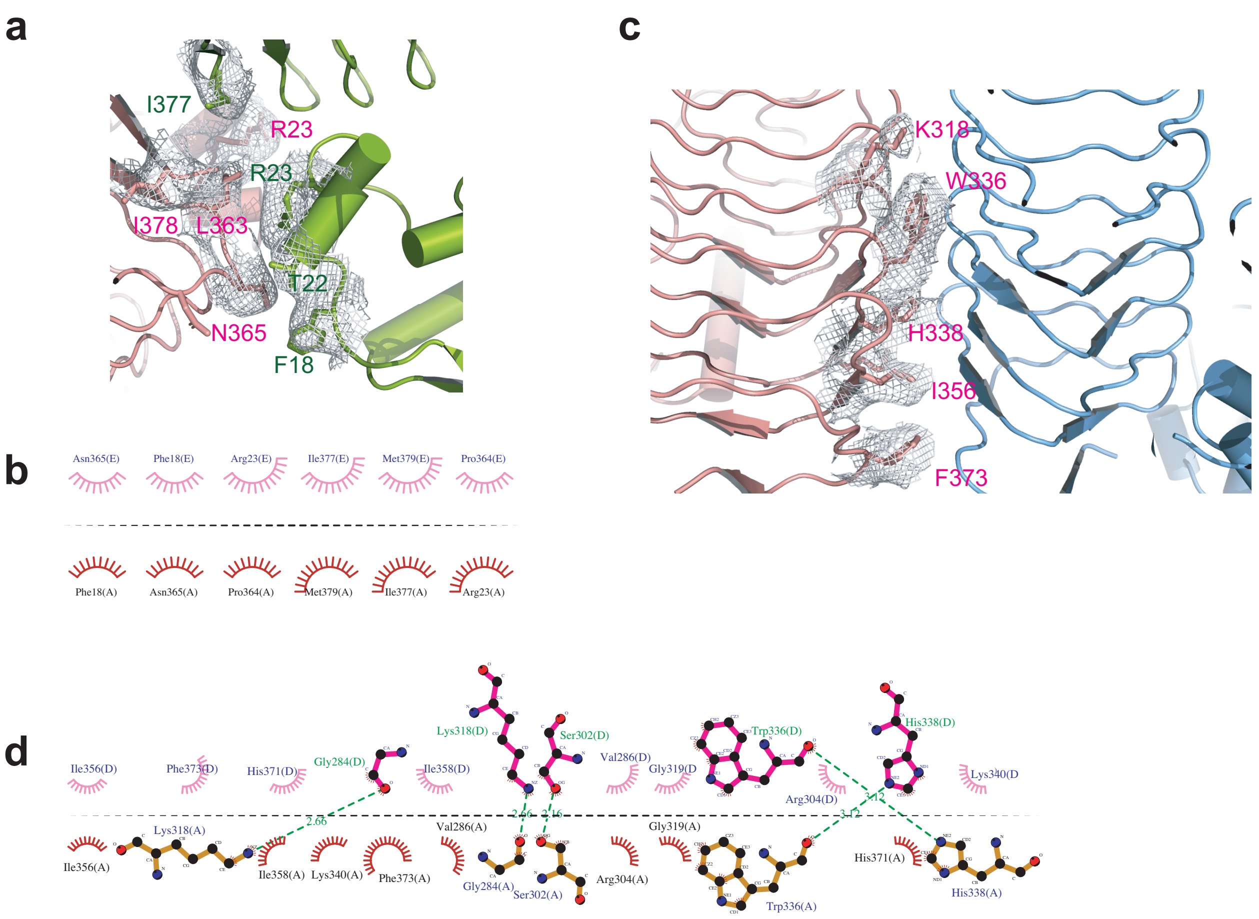
a
I377
R23
R23
I378
L363
T22
N365
F18
c
K318
W336
H338
I356
F373
b
Asn365(E)
Phe18(E)
Arg23(E)
Ile377(E)
Met379(E)
Pro364(E)
Phe18(A)
Asn365(A)
Pro364(A)
Met379(A)
Ile377(A)
Arg23(A)
d
Ile356(D)
Phe373(D)
His371(D)
Gly284(D)
Ile358(D)
Lys318(D)
Ser302(D)
Val286(D)
Gly319(D
Trp336(D)
Arg304(D)
His338(D)
Lys340(D
2.66
Ile356(A)
Lys318(A)
Ile358(A)
Lys340(A)
Phe373(A)
Val286(A)
Gly284(A)
Ser302(A)
Arg304(A)
Gly319(A)
Trp336(A)
His371(A)
His338(A)

Fig. S5

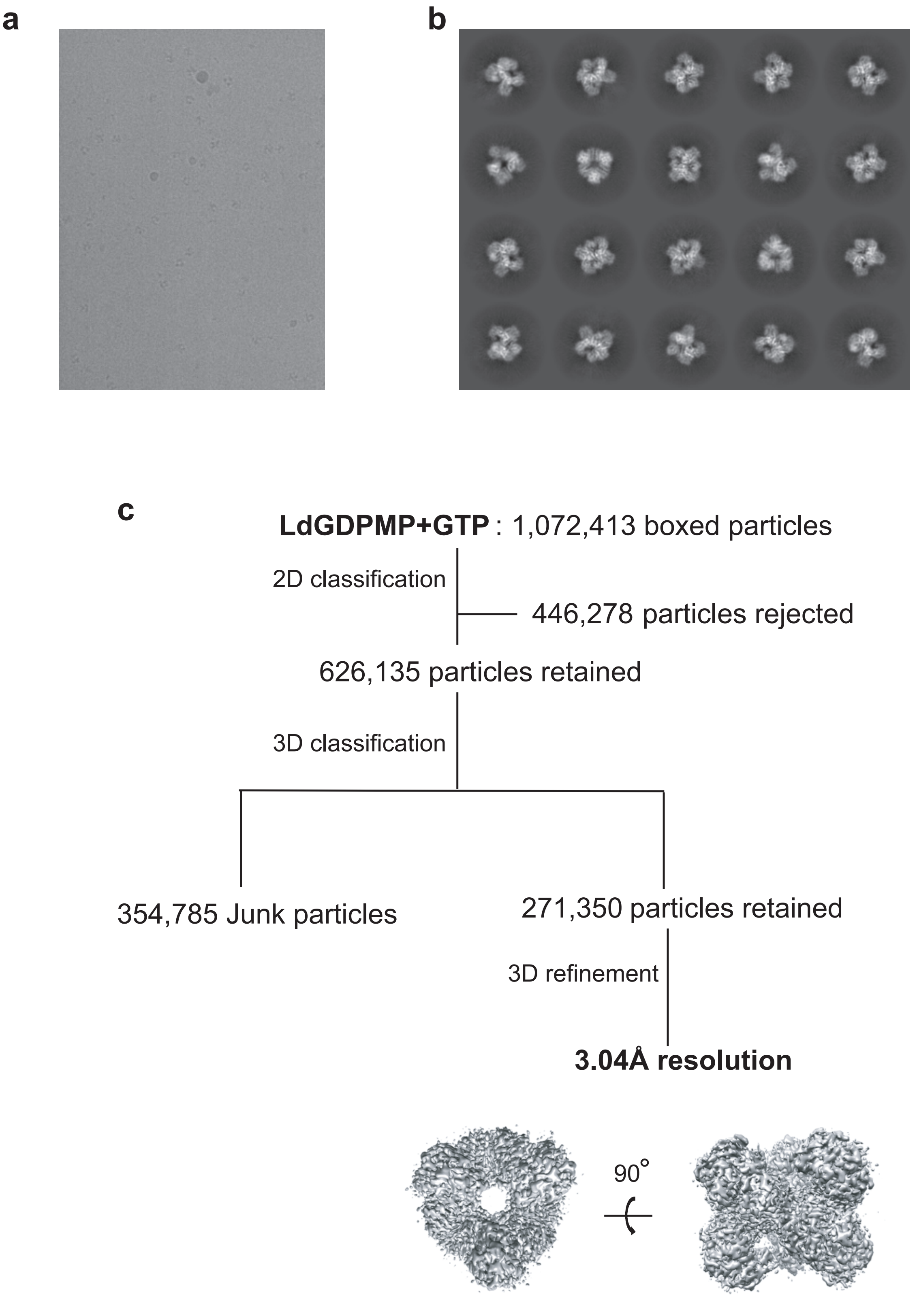
a
b
c
LdGDPMP+GTP : 1,072,413 boxed particles
2D classification
446,278 particles rejected
626,135 particles retained
3D classification
354,785 Junk particles
271,350 particles retained
3D refinement
3.04Å resolution
90°

Fig. S6

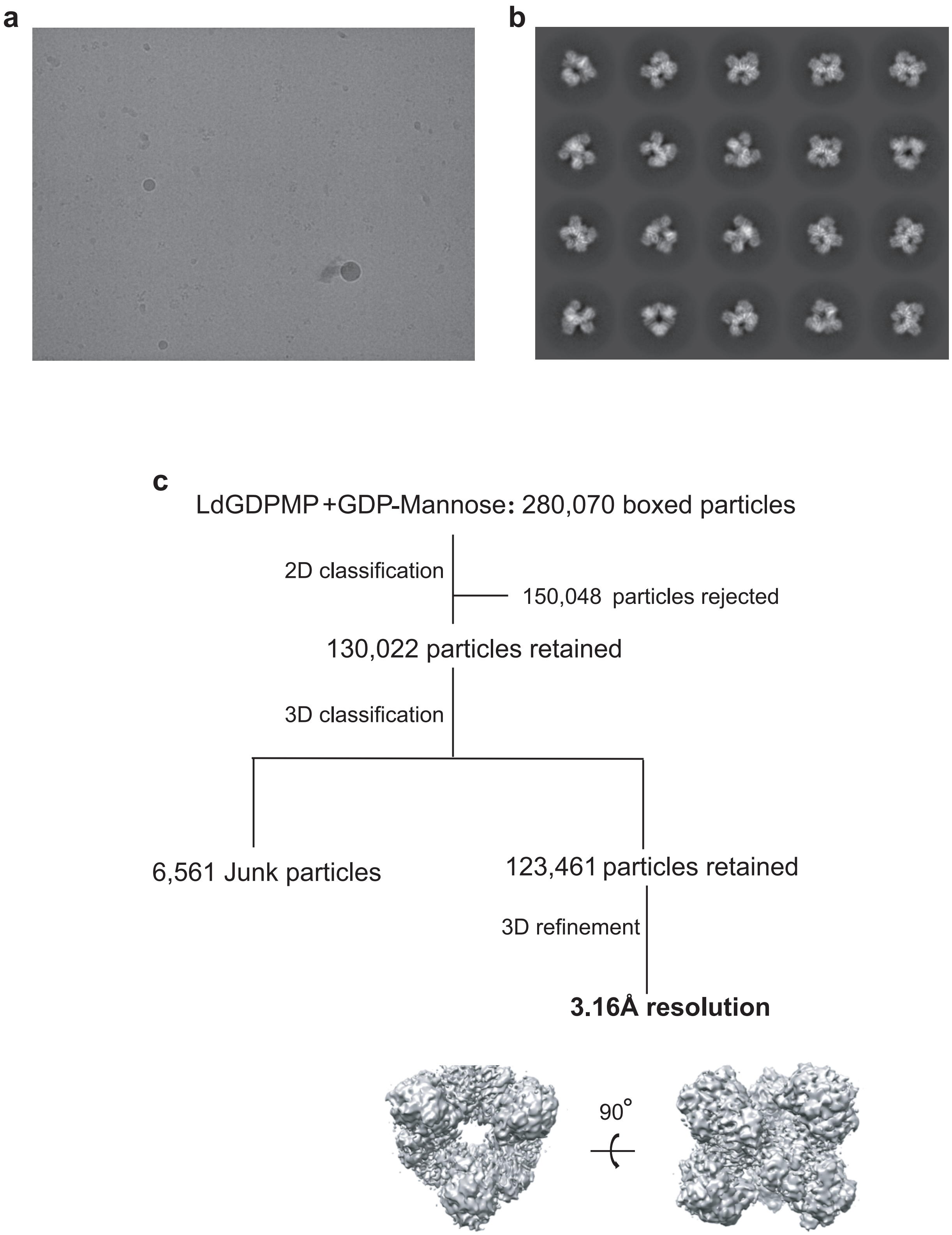
a
b
c
LdGDPMP+GDP-Mannose: 280,070 boxed particles
2D classification
150,048 particles rejected
130,022 particles retained
3D classification
6,561 Junk particles
123,461 particles retained
3D refinement
3.16Å resolution
90°

Fig. S7

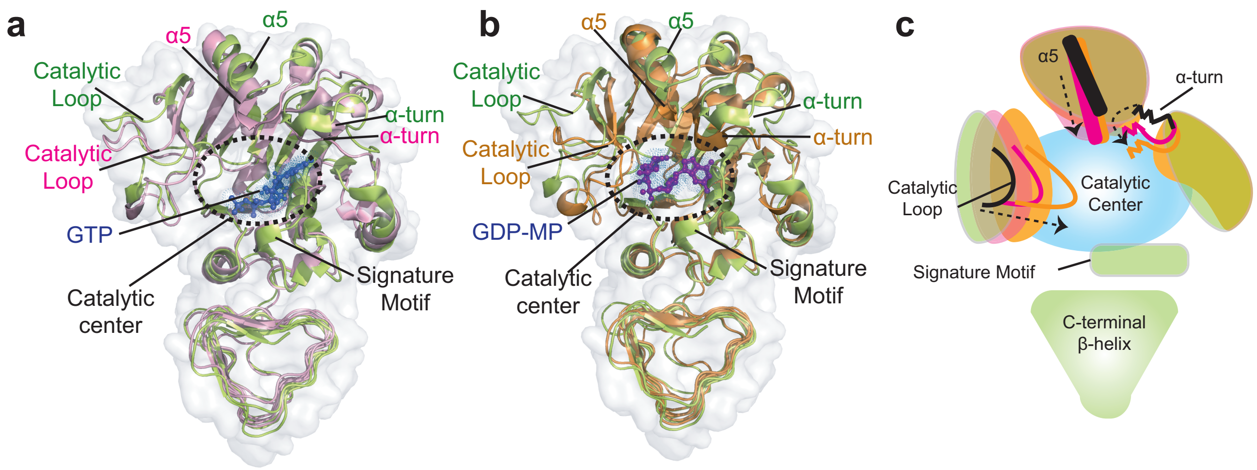

a
α5
α5
Catalytic
Loop
Catalytic
Loop
α-turn
α-turn
GTP
Catalytic
center
Signature
Motif
b
α5
α5
Catalytic
Loop
Catalytic
Loop
α-turn
α-turn
GDP-MP
Catalytic
center
Signature
Motif
c
α5
α-turn
Catalytic
Loop
Catalytic
Center
Signature Motif
C-terminal
β-helix


Fig. S8

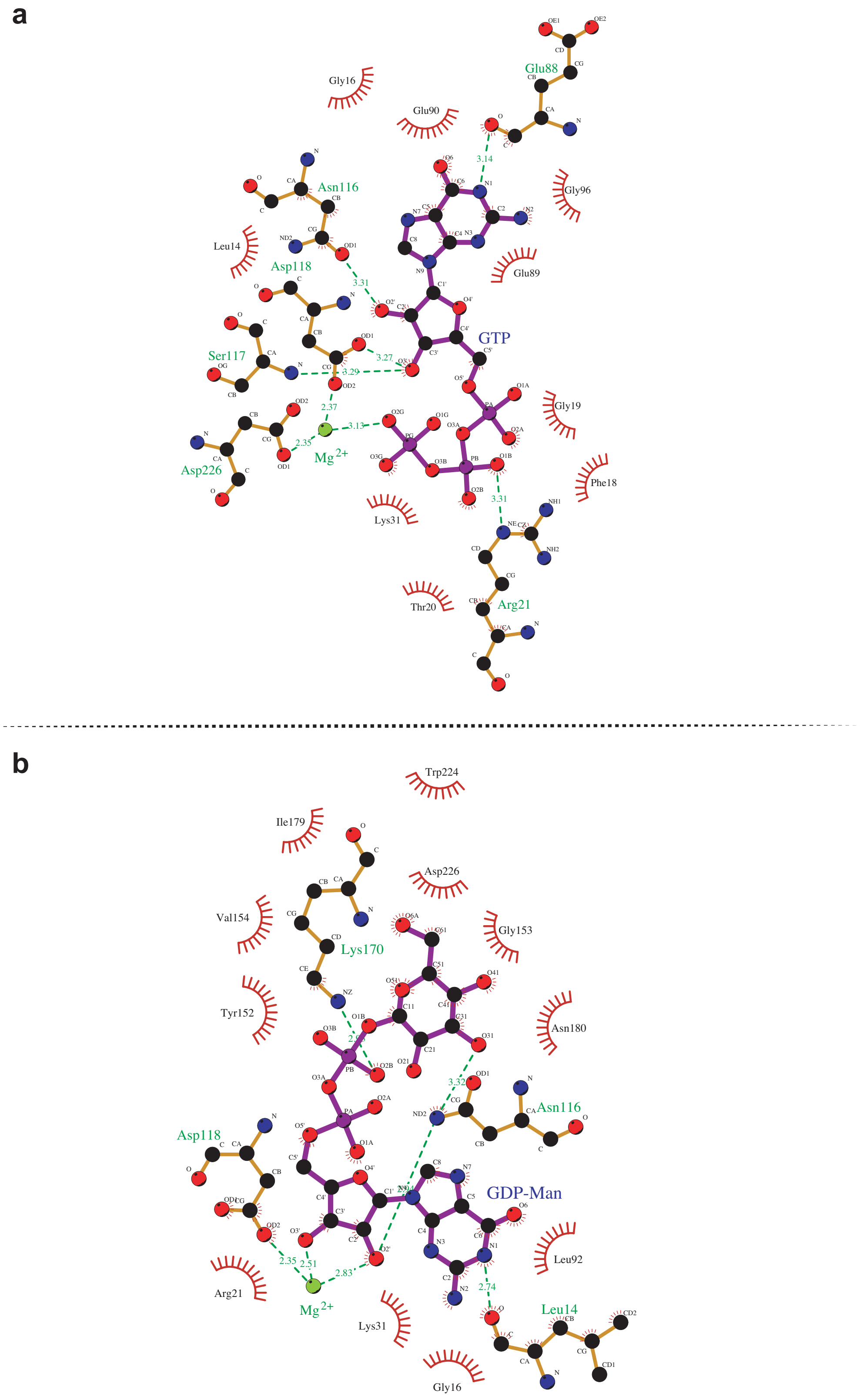
a
Gly16
Glu90
Glu88
Asn116
Gly96
Leu14
Asp118
Glu89
Ser117
GTP
Gly19
Asp226
Mg 2+
Phe18
Lys31
Thr20
Arg21
3.14
3.31
3.27
3.29
2.37
3.13
2.35
3.31
b
Trp224
Ile179
Asp226
Val154
Gly153
Lys170
Tyr152
Asn180
Asn116
Asp118
GDP-Man
Leu92
Arg21
Mg 2+
Lys31
Leu14
Gly16
3.32
2.35
2.51
2.83
2.74

Fig. S9

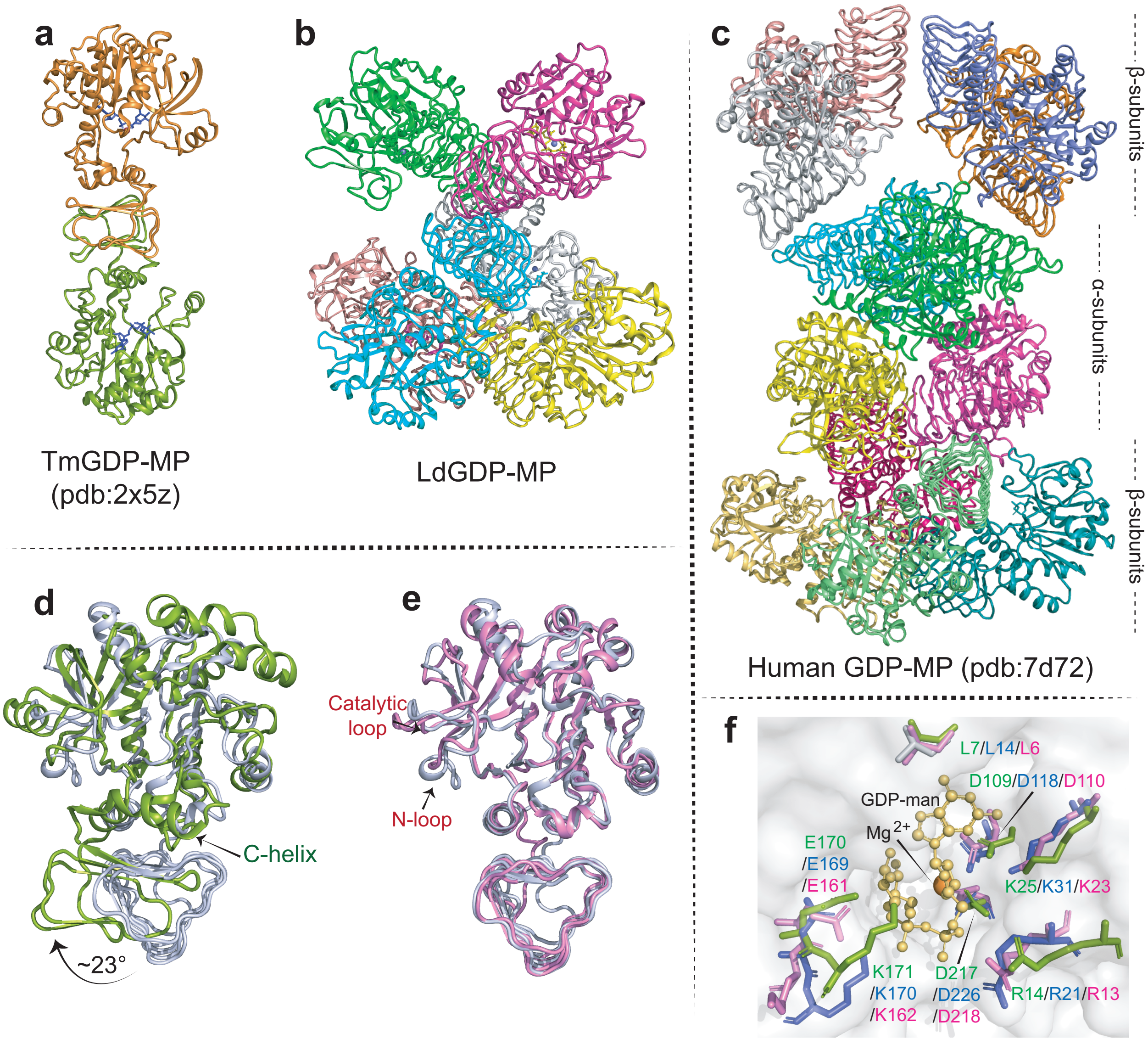
a
b
c
β-subunits
α-subunits
β-subunits
TmGDP-MP
(pdb:2x5z)
LdGDP-MP
Human GDP-MP (pdb:7d72)
d
e
f
Catalytic
loop
N-loop
C-helix
~23°
L7/L14/L6
D109/D118/D110
GDP-man
Mg 2+
E170
/E169
/E161
K25/K31/K23
K171
/K170
/K162
D217
/D226
/D218
R14/R21/R13

Fig. S10

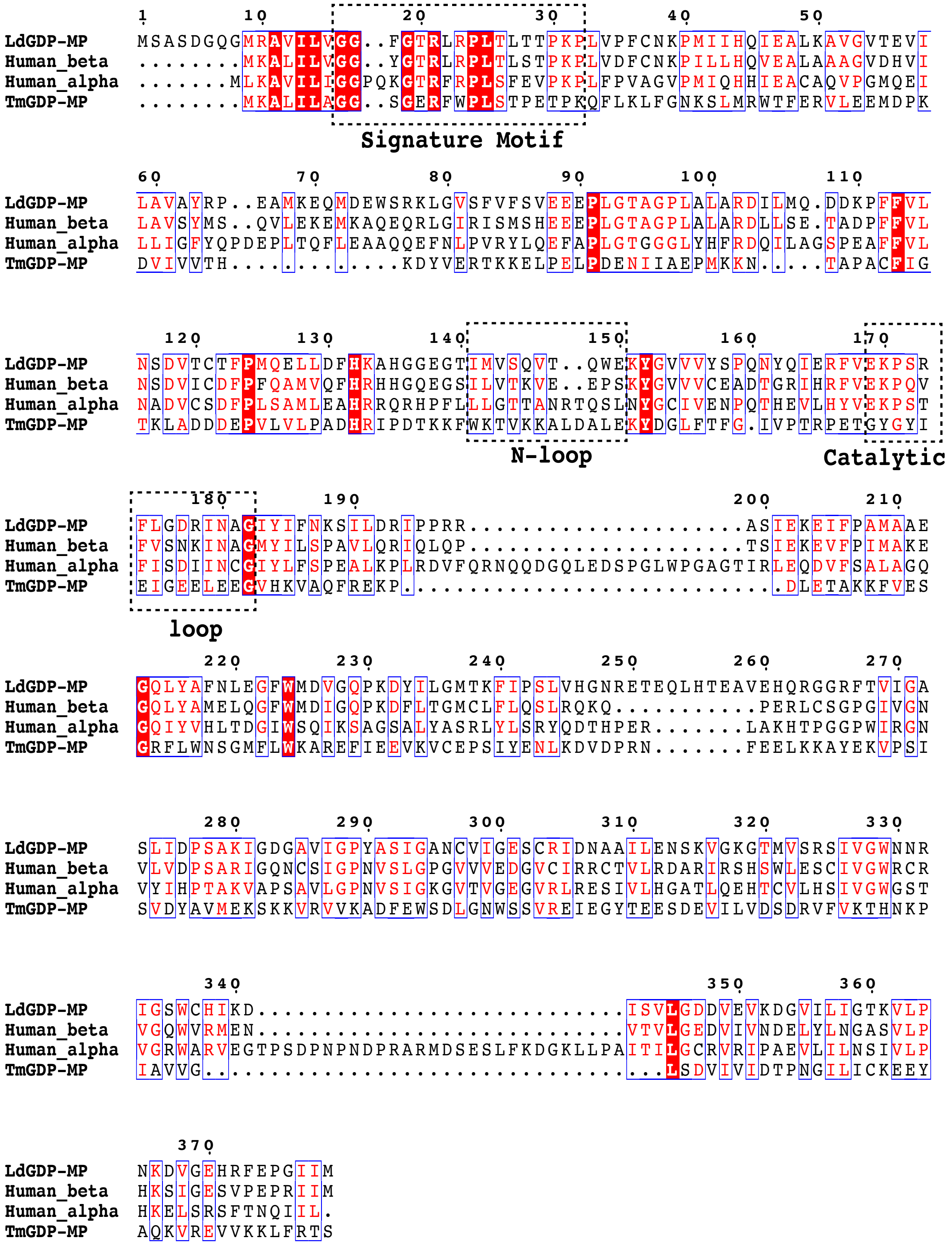
LdGDP-MP
Human_beta
Human_alpha
TmGDP-MP
Signature Motif
N-loop
Catalytic loop

Fig. S11

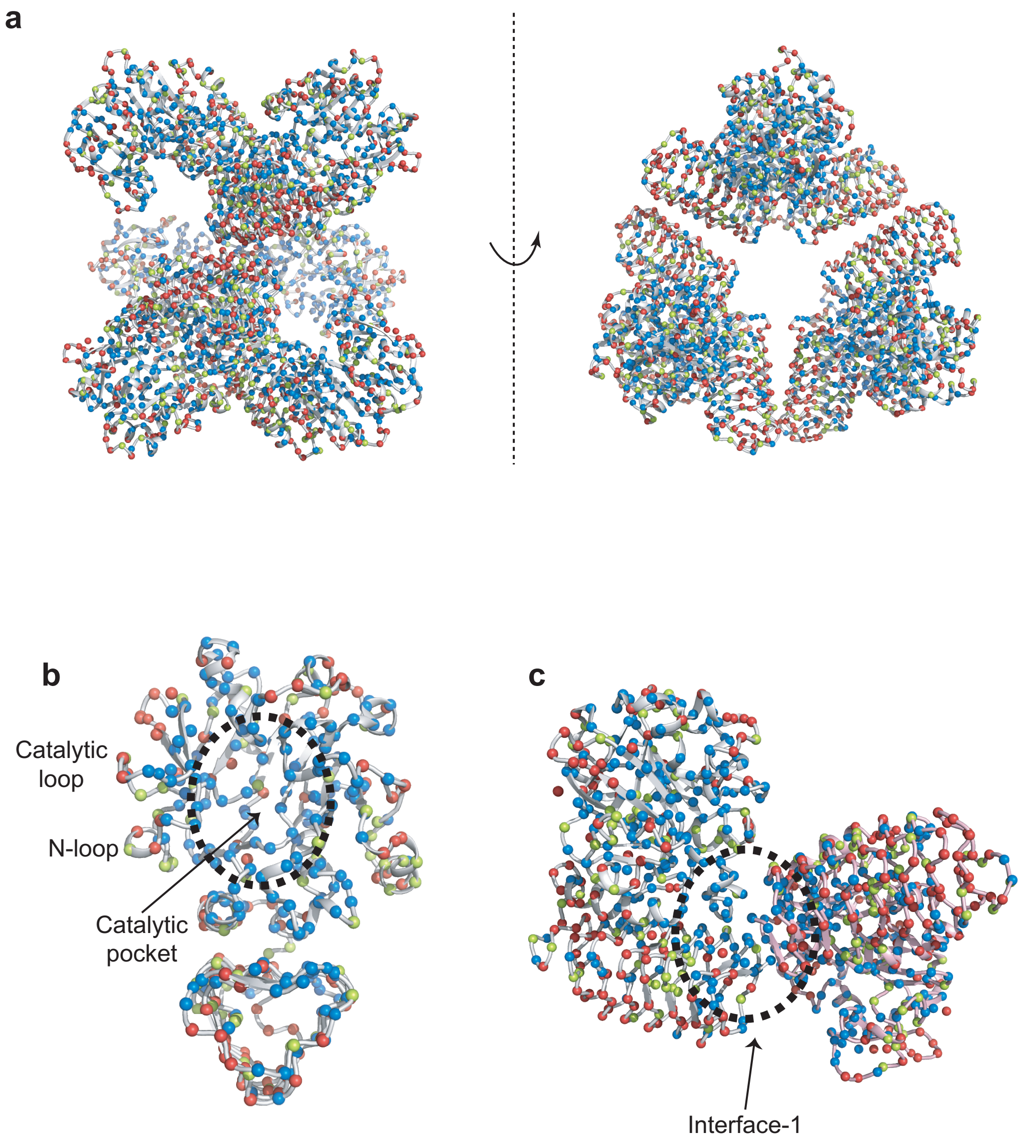


Fig. S12

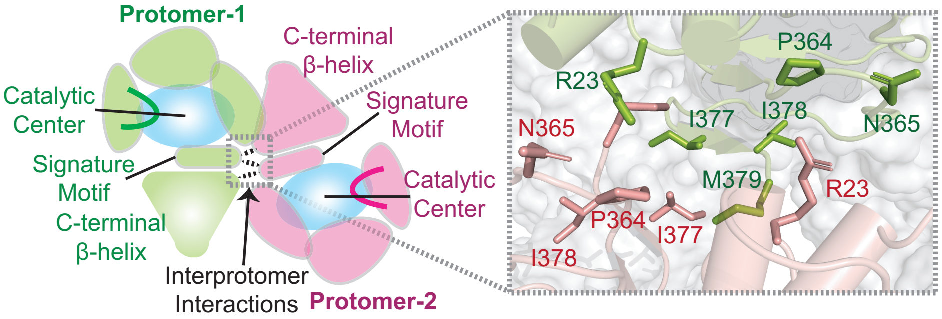
Protomer-1
Catalytic
Center
Signature
Motif
C-terminal
β-helix
C-terminal
β-helix
Signature
Motif
Catalytic
Center
Interprotomer
Interactions
Protomer-2
P364
R23
I377
I378
N365
N365
M379
R23
P364
I377
I378

Fig. S13

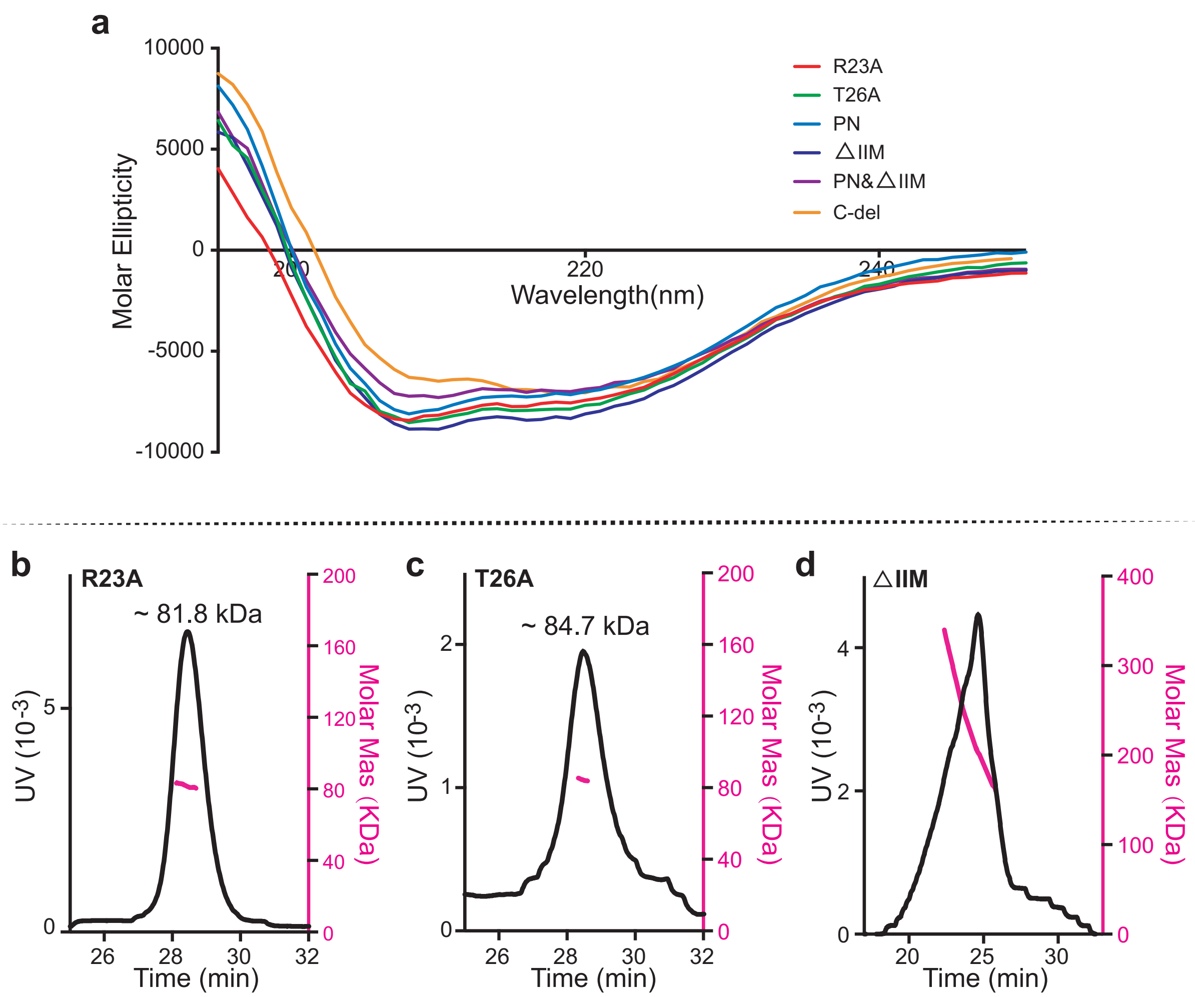


e

| | WT | R23A | T26A | △IIM |
|---|---|---|---|---|
| Oligomeric state | Hexamer | Dimer | Dimer | Mixture of oligomers |

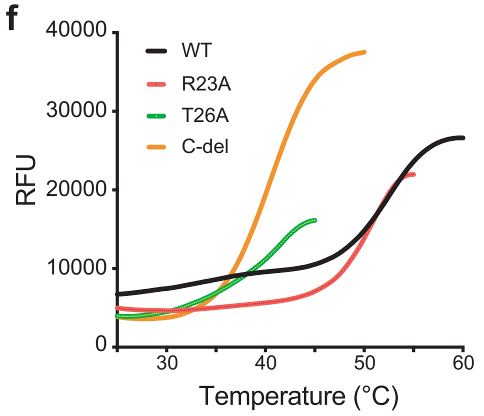


g

| | WT | R23A | T26A | C-del |
|---|---|---|---|---|
| Tm (°C) | 52.4 | 50.6 | 41.6 | 40.8 |

Fig. S14

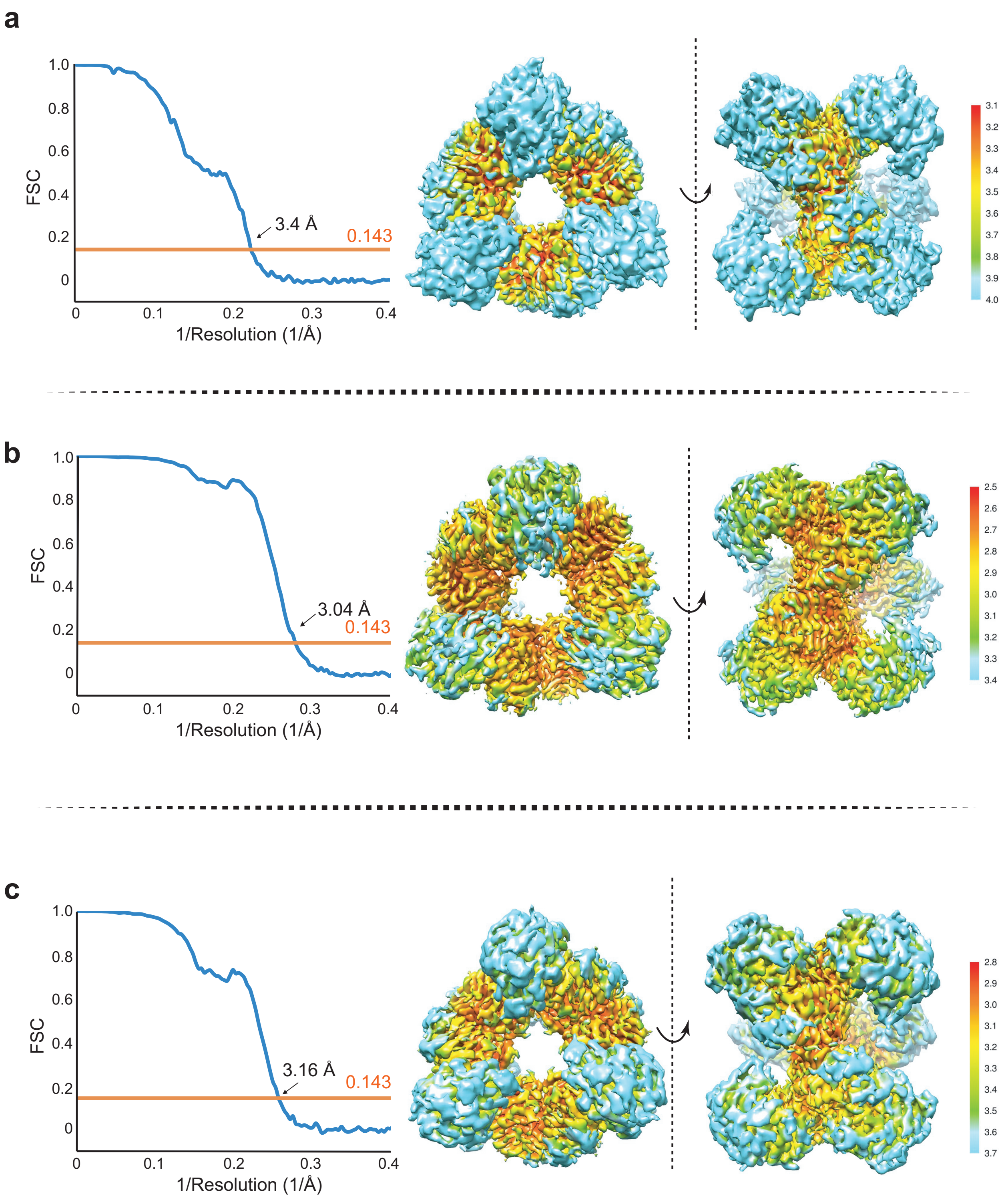
a
1.0
0.8
0.6
0.4
0.2
0
FSC
3.4 Å
0.143
0
0.1
0.2
0.3
0.4
1/Resolution (1/Å)
3.1
3.2
3.3
3.4
3.5
3.6
3.7
3.8
3.9
4.0
b
1.0
0.8
0.6
0.4
0.2
0
FSC
3.04 Å
0.143
0
0.1
0.2
0.3
0.4
1/Resolution (1/Å)
2.5
2.6
2.7
2.8
2.9
3.0
3.1
3.2
3.3
3.4
c
1.0
0.8
0.6
0.4
0.2
0
FSC
3.16 Å
0.143
0
0.1
0.2
0.3
0.4
1/Resolution (1/Å)
2.8
2.9
3.0
3.1
3.2
3.3
3.4
3.5
3.6
3.7

Fig. S15

**Table S1 Cryo-EM data collection, refinement, and validation statistics.**

| | | LDGDP_APO (PDB 7WHR) (EMDB 32509) | LDGDP_GTP (PDB 7WHS) (EMDB 32510) | LDGDP_GDP-MAN (PDB 7WHT) (EMDB 32511) |
|---|---|---|---|---|
| Magnification | | 29000 x | 29000 x | 29000 x |
| Voltage (kV) | | 300 | 300 | 300 |
| Electron exposure (e−/Å2 ) | | 50 | 50 | 50 |
| Defocus range (μm) | | -1.6 ~ -2.3 | -1.6 ~ -2.3 | -1.6 ~ -2.3 |
| Pixel size (Å) | | 1.07 | 1.07 | 1.07 |
| Symmetry imposed | | D3 | D3 | D3 |
| Chains | | 6 | 6 | 6 |
| Nonhydrogen atoms | | 17310 | 17310 | 17310 |
| Protein residues | | 2226 | 2226 | 2226 |
| Bond lengths (Å) | | 0.002 | 0.003 | 0.003 |
| Bond angles (°) | | 0.605 | 0.605 | 0.531 |
| Model-to-map fit (CC) | | 0.75 | 0.79 | 0.75 |
| Map resolution (Å) | | 3.40 | 3.04 | 3.16 |
| FSC threshold | | 0.143 | 0.143 | 0.143 |
| | | | | |
| MolProbity score | | 1.83 | 1.94 | 2.12 |
| Clashscore | | 8.11 | 8.45 | 12.31 |
| Rotamers outliers (%) | | 0.00 | 0.00 | 0.00 |
| Ramachandran parameters | Favored (%) | 94.23 | 92.13 | 90.96 |
| | Allowed (%) | 5.77 | 7.87 | 9.04 |
| | Outliers (%) | 0.00 | 0.00 | 0.00 |

**Figure legends:**

**Fig.S1. Characterization of LdGDP-MP. a.** Catalytic reaction scheme of GDP-MP. **b.** Purification of LdGDP-MP. Lanes 1 and 2 in the SDS-PAGE analysis show the protein marker and purified LdGDP-MP, respectively. **c.** SEC-MALS analysis of purified LdGDP-MP. **d** and **e.** Steady-state kinetic analysis of LdGDP-MP. Reactions were performed for 6 min at 37 °C in the presence of 2ng/μL LdGDP-MP and saturated amounts of substrates, except the concentrations of Man-1-P (**d**) or GTP (**e**) were varied. All enzyme assays were repeated at least three times and data were shown as means ± s.d.

**Fig. S2. Determination of optimal conditions of enzymatic reaction and specificities for LdGDP-MP. a.** Time courses of LdGDP-MP enzyme activity. **b-f.** Determination of the optimal conditions for enzymatic reaction: metal ion cofactors (**b**), $Mg^{2+}$ concentration (**c**), pH value (**d**), substrate specificities (**e**), and temperature (**f**). All enzyme assays were repeated at least three times and data were shown as means ± s.d.

**Fig. S3. Cryo-EM analysis of Apo LdGDP-MP. a.** Representative cryo-EM micrograph. **b.** Subsets of representative, reference-free 2D class averages. **c.** Data processing workflow.

**Fig. S4. Secondary structure components of LdGDP-MP. a.** Ribbon diagrams of a LdGDP-MP monomer with secondary components highlighted and labeled. **b.** Diagrams of domain organization of LdGDP-MP. The β strands, α helices and the C-terminal β-helix are represented as arrows in green, cylinders in pink, and arrows in orange, respectively.

**Fig. S5. Detailed inter-protomer contacts of LdGDP-MP. a.** Interface-1 with real density map shown in mesh. Only representative interface residues are shown for simplicity purpose. **b.** Interface-1 analysis using the program of Ligplot. **c.** Interface-2 with real density map shown in mesh. Only representative interface residues in one subunit are shown for simplicity purpose. **d.** Interface-2 analysis using the program of Ligplot.

**Fig. S6. Cryo-EM analysis of LdGDP-MP in complex with GTP. a.** Representative cryo-EM micrograph. **b.** Subsets of representative, reference-free 2D class averages. **c.** Data processing workflow.

**Fig. S7. Cryo-EM analysis of LdGDP-MP in complex with GDP-Man. a.** Representative cryo-EM micrograph. **b.** Subsets of representative, reference-free 2D class averages. **c.** Data processing workflow.

**Fig. S8. Catalytic pocket delineation for LdGDP-MP.** Cryo-EM structures of LdGDP-MP in complex with GTP (**a**) and GDP-Man (**b**) with magnified views of substrate or product binding pocket. The key elements involved in catalysis are shown. **c.** Cartoon depiction of drastic structural changes of LdGDP-MP upon substrate binding or production formation. Green: Apo LdGDP-MP; Pink: LdGDP-MP in complex with GTP; Orange: LdGDP-MP in complex with the reaction product of GDP-Man.

**Fig. S9. Detailed analysis of interactions of substrate and product with LdGDP-MP using the program of Ligplot. a.** GTP. **b.** GDP-Man.

**Fig. S10. Structural comparisons of GDP-MPs from various species. a.** Dimeric structure of the bacterial TmGDP-MP (pdb: 2x5z). **b.** Hexameric organization of LdGDP-MP. **c.** Structure of heterododecameric human GDP-MP (pdb 7d72). **d.** Overlay of the structure of the bacterial TmGDP-MP (green) with LdGDP-MP (grey). **e** Overlay of the structure of the human GDP-MP complex (pink) with LdGDP-MP (grey). Note the structural variations at the catalytic loop and N-loop regions. **f.** Superimposed structures of the catalytic centers of TmGDP-MP (green), LdGDP-MP (blue) and human GDP-MP (pink). High structure similarities in reaction centers suggest the catalytic mechanism for these three GDP-MP species are essentially identical.

**Fig. S11. Amino acid sequence alignments of GDP-MPs from various species.** The sequence is numbered according to the GDP-MP from *L. donovani* (LdGDP-MP) and the regions of canonical signature motif, N-loop and catalytic loop are labeled.

**Fig. S12. Amino acid conservation view of LdGDP-MP with human GDP-MP in the context of structure.** The identical, conserved and non-conserved amino acids are depicted by spheres in colors of blue, green and red, respectively. **a-c.** Conservation view of LdGDP-MP with human GDP-MP in the context of hexameric structure (**a**), monomer structure (**b**) and the inter-protomer contacts (**c**). Note that the conserved amino acids are largely clustered around the catalytic pocket, while the non-conserved residues are distributed in the peripheral regions, especially at the N-loop and catalytic loop.

**Fig. S13. Detailed contacts in the interface-1 region.**

**Fig. S14. Biophysical characterizations of LdGDP-MP mutants. a.** Circular dichroism (CD) spectroscopy analysis of various LdGDP-MP mutants. The mutants are labeled. PN: P364R/N365R; ∆IIM : deletion of residues I377, I378 and M379; PN&∆IIM: combination mutations of P364R/N365R and deletion of residues I377, I378 and M379; C-del: deletion of the entire C-terminal domain (residues 252-379). **b-e.** SEC-MALS analysis of LdGDP-MP mutants. SEC-MALS profiles are demonstrated for R23A (b), T26A (c) and ∆IIM with calculated molar mass shown, and oligomeric states are summarized in (**e**). **f-g.** Thermal shift assays for LdGDP-MP and mutants. The thermal stabilities of wild type LdGDP-MP and mutants were analyzed by measuring SYPRO Orange dye fluorescence over a temperature ranging from 25 to 60 °C using a real-time PCR thermocycler. **f.** Representative unfolding curves. **g.** Derived melting temperatures. RFU: Relative fluorescence unit. The lower values of melting temperature for the mutants indicate structure instability.

**Fig. S15. Validation of cryo-EM structures**. **a-c.** Apo LdGDP-MP, LdGDP-MP in complex with GDP and GDP-Man, respectively. Left: Gold-standard FSC plots generated by cryoSPARC; Right: Final local resolution estimation of the cryo-EM maps.
